# New perspectives in biliary tract cancers

**DOI:** 10.1016/j.esmogo.2024.100092

**Published:** 2024-08-30

**Authors:** T.O. Goetze, C. Roderburg, F.W. Friedrich, J. Trojan

**Affiliations:** 1 Krankenhaus Nordwest, Institute of Clinical Cancer Research, University Cancer Center, Frankfurt; 2 Clinic for Gastroenterology, Hepatology and Infectious Diseases, Medical Faculty of Heinrich Heine University Düsseldorf, University Hospital Düsseldorf, Düsseldorf; 3 AstraZeneca GmbH, Hamburg; 4 Department of Internal Medicine 1 and University Cancer Center, University Hospital Frankfurt, Goethe University, Frankfurt am Main, Germany

**Keywords:** biliary tract cancer, cholangiocarcinoma, treatment, chemotherapy, immunotherapy, targeted therapy

## Abstract

Biliary tract cancer (BTC) is a rare but highly lethal malignancy. Despite recent advances in diagnosis and treatment, the overall prognosis remains dismal, with a median survival of <1 year in most cases. This highlights an urgent medical need for better treatment options, especially in the area of systematic treatments.

This review aims to give a concise overview of the current available treatment options for BTC, including a short summary of longstanding therapeutic approaches such as surgery, interventional techniques, radiotherapy, chemotherapy, and chemoradiotherapy. Special emphasis is placed on genetic alterations and treatment advances with immunotherapy in combination with chemotherapy, however, including current trials on new immunotherapeutic drugs. Furthermore, the recent recommendation by international guidelines to use durvalumab plus the combination of gemcitabine and cisplatin as a first-line treatment in the advanced setting is highlighted and the evidence supporting this recommendation is explored. Moreover, this review looks at genetic alterations which can be used as targets for immunotherapy, especially isocitrate dehydrogenase 1/2 (*IDH1/2*), fibroblast growth factor receptor 2 (*FGFR2*), and human epidermal growth factor receptor 2 (*HER2/neu*). Upcoming biomarkers such as microRNAs (miRNAs and especially miR-221) can possibly facilitate the choice of the appropriate treatment regimen in the future.

We conclude that there is a lot of recent development in the area of biomarker-driven targeted therapies and immunotherapies for BTC, which could consequently bring major benefits to patients’ treatment outcomes and quality of life.

## Highlights

• Review of treatments for biliary tract cancer, especially in the advanced stage.
• Special focus on genetic alterations and advances in immunotherapies until now.
• Review of durvalumab and gemcitabine plus cisplatin as first-line therapy.
• Outlook on current trials and new developments of immunotherapeutic drugs.
• Biomarker-driven targeted therapies and immunotherapies promise to improve outcomes.

## Graphical abstract

**Figure:**
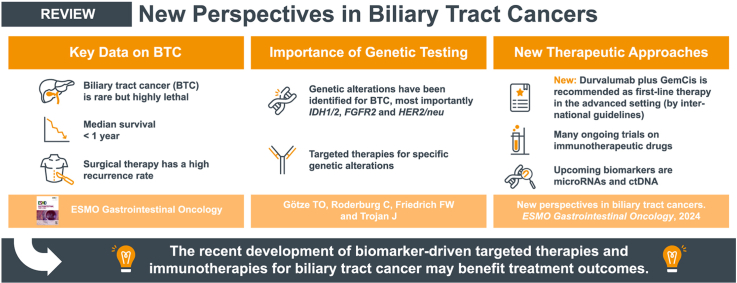

## Introduction

Biliary tract cancers (BTC) are malignancies arising from the biliary tree that includes the gallbladder, bile ducts, and ampulla of Vater. BTCs are low in incidence, but high in mortality rate with a poor prognosis.^1^ With a 5-year survival rate of only 10%-17%, BTC is one of the most aggressive and treatment-resistant cancers, having the second worst prognosis after pancreatic cancer.^2^

Radical resection is still the only curative treatment option, but recurrence rates are high and most patients (>80%) present with advanced, unresectable disease.^3^ Adjuvant therapy with capecitabine was implemented as standard of care for patients with curatively resected BTC in the EU and the USA. In the advanced setting, gemcitabine plus cisplatin (GemCis) has been the standard of care first-line therapy approach for more than a decade.

Recently, the treatment landscape in the advanced setting changed drastically due to the approval of the combination of the immune checkpoint inhibitor (ICI) durvalumab with chemotherapy as a novel first-line treatment option.^4^^,^^5^ Durvalumab or pembrolizumab in combination with GemCis is now considered the standard of care for first-line treatment of BTC in the advanced setting.^6^^,^^7^ In the second-line setting, targeted therapies are frequently used as 40%-50% of patients harbour targetable genetic alterations.^8^, ^9^, ^10^ Targetable genetic alterations are defined as oncogenic driver alterations that can be targeted by treatment, including with therapeutic agents still being under investigation or approved in other tumour types.^9^ The ESMO Scale for Clinical Actionability of Molecular Targets (ESCAT) provides a systematic framework to rank those molecular targets.^6^

Considering the recent developments regarding therapeutic options for BTC, especially in the advanced setting, this review gives a short overview of relevant biomarkers, and treatment options for BTC. Special emphasis is placed on recent developments of immunotherapy in the advanced BTC setting and on the analyses of genetic alterations and their impact on treatment.

## Biomarkers and genomic alterations

Analyses of biomarkers and genomic alterations are not only important for diagnosis, but also for the estimation of the patients’ prognosis and initial treatment decisions. At the time of writing, no bile biomarkers for diagnosis, monitoring, or staging of BTC are in clinical use.^11^ Several cell-free non-coding RNAs (especially miRNA such as miR-221), however, show promising results as potentially new biomarkers for BTC.^12^^,^^13^

Furthermore, circulating tumour DNA (ctDNA) analysis may be used to identify patients with genetic alterations.^12^ Genetic alterations are primarily linked to tumour location as highlighted by Nakamura et al.^14^ Detailed information on specific genetic alterations can be found in Table 1.

**Table 1:** Cancer types/locations and association with genetic alterations.

Cancer type/location	Genetic alteration
iCCA	*IDH1/2*: isocitrate dehydrogenase 1/2 mutations *EPHA2*: EPH receptor A2 *BAP1*: BRCA1 associated protein 1 mutations *FGFR2*: fibroblast growth factor receptor 2 fusions and mutations *BRAF*^*V600E*^: v-Raf murine sarcoma viral oncogene homolog B mutations^14^^,^^15^ *HER2/neu*: human epidermal growth factor receptor 2 mutations and amplifications^16^ *ARID1A*: AT-rich interaction domain 1A *BRCA*: breast cancer gene *KRAS G12C*: mutation of KRAS protein *MSI*: microsatellite instability
eCCA	*ELF3*: ETS transcription factor 3 *ARID1B*: AT-rich interaction domain 1B mutations *PRKACA/PRKACB*: protein kinase cAMP-activated catalytic subunit alpha and beta fusions^14^^,^^15^ *HER2/neu:* human epidermal growth factor receptor 2^16^ *KRAS G12C*: mutation of KRAS protein *MSI*: microsatellite instability
GBC	*EGFR*: epidermal growth factor receptor *ERBB3*: erb-b2 receptor tyrosine kinase 3 *PTEN*: phosphatase and tensin homolog promotor mutations *CDKN2A/B*: cyclin dependent kinase inhibitor 2A/B *HER2/neu*: amplification, overexpression, or both^14^^,^^17^ *KRAS G12C*: mutation of KRAS protein *MSI*: microsatellite instability

eCCA, extrahepatic cholangiocarcinoma; GBC, gallbladder carcinoma; iCCA, intrahepatic cholangiocarcinoma.

Additionally, Lowery et al. ^8^ showed that isocitrate dehydrogenase 1 (*IDH1*), AT-rich interaction domain 1A (*ARID1A*), breast cancer 1 (BRCA1)-associated protein 1 (*BAP1*), and tumour protein p53 (*TP53*) were the most commonly altered genes, along with fibroblast growth factor receptor 2 (*FGFR2*) fusions. Mutual exclusivity of genomic alterations was also shown for *TP53:IDH1*, *IDH1:* Kirsten rat sarcoma virus (*KRAS*), *TP53:BAP1*, and *IDH1:FGFR2*. Crucially, in patients on chemotherapy with locally advanced and metastatic disease, cyclin-dependent kinase inhibitor 2A/B (*CDKN2A/B*) and erb-b2 receptor tyrosine kinase 2 (*ERBB2*) alterations were linked to reduced survival and time to progression.^8^ These findings suggest that BTC should be treated according to the tumour’s anatomic location and its genetic alterations.

Risk factors and their associated diseases should be taken into account as well, however, e.g. liver fluke infections change the genetic landscape of cholangiocarcinoma (CCA) drastically.^18^ While liver fluke-positive CCAs exhibit *TP53* mutations and *ERBB2* amplifications, liver fluke-negative CCAs present with either epigenetic mutations (*IDH1/2*, *BAP1*) and *FGFR2* fusions or programmed cell death protein 1/programmed death-ligand 2 (*PD-1/PD-L2*) mutations and high copy number alterations.^18^ These results point to a mixture of genetics, diseases and carcinogens being the drivers behind the genesis of different distinct CCA subtypes.

### *FGFR2* and *IDH1/IDH2*

*FGFR2* fusions and *IDH1*/*IDH2* mutations are genetic alterations in intrahepatic cholangiocarcinoma (iCCA) for targeted therapies which have already shown effectiveness.^12^^,^^19^ The diagnostically used *FGFR2* fusions and *IDH1/2* mutations are exclusive to iCCA of the small-duct type.^20^^,^^21^ By contrast, the large-duct type is characterized by *KRAS* and *TP53* mutations.^22^ In 10%-17% of iCCA patients *FGFR2* fusions are present, whereas 10%-20% show *IDH* mutations.^10^^,^^23^, ^24^, ^25^

*FGFR2* is a protein that constitutes the cell surface tyrosine kinase receptor for fibroblast growth factor. Overexpression of *FGFR2* fusion proteins is generally associated with higher cell motility, elevated cell proliferation, increased cell differentiation, and modified cell morphology.^20^^,^^26^^,^^27^

*IDH1/IDH2* mutations are the most common alterations to target. According to a review, mutated *IDH1* was present in 13.1% [95% confidence interval (CI) 12.1% to 14.2%] of iCCA patients and 0.8% (95% CI 0.4% to 1.5%) of extrahepatic cholangiocarcinoma (eCCA) patients.^28^ Analyses of *IDH* mutations’ molecular genesis in CCA reveal specific features concerning mRNA, copy number, DNA methylation, high mitochondrial, low chromatin modifier gene expression, methylation of the *ARID1A* promoter, and low *ARID1A* expression.^29^ These findings indicate that other liver tumours with *IDH* mutations could be similar to CCA and be addressed through *IDH* inhibiting medication.

### Further genetic alterations

Hepatic cells that can be reprogrammed into cancer stem cells and cells of origin like hepatic progenitor cells and progenitor-like cells are important factors of oncogenesis and could consequently lead to improved targeted therapies for CCA patients.^20^^,^^30^ Moreover, other genetic alterations with implications for BTC are the human epidermal growth factor receptor 2 (*HER2/neu*), high microsatellite instability/mismatch repair-deficient (*MSI-H/dMMR*), neurotrophic receptor tyrosine kinase (*NTRK*), and v Raf murine sarcoma viral oncogene homolog B (*BRAF*^*V600E*^).

*HER2/neu* is a well-established oncoprotein, which is successfully used as a target in precision oncology. While other mutations are primarily linked to tumour location, *HER2/neu* amplification, overexpression, or both are found in a wider range of BTC subtypes: *HER2/neu* is most frequently linked to gallbladder carcinoma (GBC), followed by eCCA, and has more recently also been detected in iCCA. The incidence varies depending on the study: 19% versus 52% for GBC, 17% versus 18% for eCCA, 5% versus 30% for iCCA (MyPathway^17^ versus HERIZON-BTC-01^16^). Moreover, apart from targeting *HER2/neu* amplification and overexpression, the therapeutic approach of targeting activating somatic *HER2/neu* mutations is being explored. In the phase II basket trial SUMMIT, the most common *HER2/neu* mutations were S310F (*n* = 11; 48%) and V777L (*n* = 4; 17%). In patients with cancer progression, loss of amplified *HER2/neu* S310F and acquisition of multiple previously undetected oncogenic co-mutations were identified.^31^

*MSI-H/dMMR* cancers occur in up to 2.1% of all BTC patients, but are present in up to 18.2% of iCCA patients and can be addressed via targeted therapy.^32^ It was already verified that patients with *MSI-H/dMMR* are responsive to therapy with ICIs.^32^

*NTRK* gene fusions are rare alterations (present in <1% of all BTC). Inhibitors of *NTRK*, however, are very efficacious and safe agents that can be administered for this rare genetic alteration.^33^

*BRAF*^*V600E*^ mutations are present in 5%-7% of all BTC.^34^ Patients harbouring this mutation show decreased overall survival (OS) and increased chance of lymph node involvement, which makes targeting *BRAF*^*V600E*^ mutations important.^34^

Standardized molecular testing for *FGFR2*, *IDH1*, *HER2/neu*, *BRAF*^*V600E*^, and *NTRK* via next-generation sequencing has already been thoroughly reviewed and is recommended before initiating first-line treatment by the European Society for Medical Oncology (ESMO) guidelines.^6^^,^^21^ The German S3 guideline recommends molecular testing for patients with an Eastern Cooperative Oncology Group (ECOG) score of 0-2 after failure of first-line therapy at the latest and in the palliative setting.^35^ Recent studies have shown that 50% of patients with genomic alterations are already receiving a targeted therapy that has been approved or is currently being investigated in clinical trials.^36^^,^^37^ Building on this, the German Onkopedia guideline (published by the German Society for Haematology and Oncology, DGHO), recommends treating patients within the framework of clinical trials whenever possible.^38^

## Treatment

As discussed earlier, the presence of specific genetic alterations is crucial for the success of targeted, individualized treatment decisions in BTC. Notwithstanding the recent therapeutic advances such as targeted therapies and ICIs, chemotherapy is still part of the backbone of many treatment regimens for BTC. Other possible treatment strategies include surgery and interventional techniques, as well as radiotherapy and chemoradiotherapy. The individual therapeutic decision is determined in the context of a multidisciplinary tumour board depending on the tumour stage and other diagnostic findings such as genetic alterations.^38^

### Localized and locally advanced stage

#### Surgery

For bile duct carcinomas (iCCA and eCCA) in stages I-III, surgical excision with the goal of complete tumour resection (R0) is currently the only curative treatment option.^35^^,^^39^ Recurrence rates for R1 resections—especially in perihilar cholangiocarcinoma (pCCA)—are high.^6^ Five-year survival rates of only 20%-50% are achieved, depending on stage and selection.^38^ In iCCA with initially unresectable tumours, it is possible to first perform downsizing with neoadjuvant chemotherapy.

According to the German S3 guideline, surgery is also recommended for GBC if resection (R0) appears possible and the tumour shows no distant metastasis.^35^ A study has demonstrated that nodal-negative, re-resected T1 patients had a 5-year survival rate of 75% and even T2 and T3 patients showed improved survival rates compared with nodal-positive subjects.^40^ Accordingly, lymph node involvement is a major negative predictive factor in stage I-III GBC. Complete surgical resection should be carried out from stage T1b onwards and even late T stages are no contraindication for surgery.^35^

#### Interventional techniques (ablation/transarterial radioembolization/transarterial chemoembolization)

Ablation may be an option for unresectable and recurrent iCCA,^41^^,^^42^ especially for patients who are deemed surgery ineligible, i.e. because of advanced age and/or comorbidities.^42^ Combination of immune checkpoint inhibition and ablation has proven safe in patients with ECOG score of 0-1.^43^ No significant changes in efficacy [OS, progression-free survival (PFS)], however, were observed.

Transarterial radioembolization (TARE; synonymous: selective internal radiation therapy, SIRT) uses radioisotopes like yttrium-90 (^90^Y) resin microspheres to treat locally advanced stages of unresectable iCCA as an alternative or after failure of systemic therapy. Recent data suggest that disease control is high and downstaging was effective.^44^^,^^45^ Sufficient randomized data, however, are currently lacking. An ongoing trial investigating the additional benefit of SIRT is IMMUWHY comparing durvalumab alone versus durvalumab plus tremelimumab—both in combination with SIRT.^46^

Transarterial chemoembolization (TACE) is primarily used in patients with advanced BTC, with reasonable efficacy and safety.^47^^,^^48^ In small, retrospective studies, the addition of TACE to chemotherapy led to similar or inferior outcomes regarding OS.^49^ A meta-analysis comparing TACE with TARE found no significant difference in patients’ outcomes, but a trend towards less adverse events in favour of TARE.^50^

#### Radiotherapy and chemoradiotherapy

In retrospective studies, adjuvant radiotherapy significantly improved OS for BTC patients compared with patients with surgical excision alone.^51^, ^52^, ^53^ Adjuvant chemoradiation shows even greater benefits in OS.^52^, ^53^, ^54^ After finishing adjuvant capecitabine therapy, radiotherapy might be considered in patients with R1 resection of GBC, distal cholangiocarcinoma (dCCA), or pCCA.^6^ Patients treated with neoadjuvant chemoradiation for tumour downsizing exhibit prolonged OS and similar results in surgical morbidity.^54^

Stereotactic body radiation therapy (SBRT) is used in iCCA patients with small lesions, where complete resection is deemed not possible. In a recent study, median OS (mOS) was 22.5 months, 1-year OS was 69.7%, and 2-year OS was 46.5%.^55^ The German PEARLDIFER study is currently recruiting and assessing whether pemigatinib in combination with SBRT can lead to organ preservation in locally advanced iCCA with *FGFR2* fusions or rearrangements.^56^ The randomized phase II trial ABC-07 showed a longer mOS and better primary tumour control with SBRT in addition to GemCis compared with GemCis alone.^57^ Interestingly, in the ABC-07 study in the experimental arm, patients treated with SBRT received only six instead of eight cycles of chemotherapy.

#### Role of neoadjuvant-perioperative and adjuvant therapy

Tumour downsizing through chemoradiotherapy (GemCis) for locally advanced and lymph node-positive stages may have a role before radical surgery. Due to a high 3-year recurrence rate of up to 80% after resection with curative intent, neoadjuvant-perioperative and adjuvant concepts of therapy are discussed as an important feature of BTC therapy regimens.^6^^,^^56^^,^^58^

Most trials studying neoadjuvant-perioperative and adjuvant therapy are retrospective analyses with limited validity. Multiple clinical trials are now conducted to further assess this field.^59^ The phase III study ACTICCA-1 compared GemCis with standard of care (currently capecitabine) after curative intent resection of CCA, but results are still pending.^60^ Two recent trials (BCAT, PRODIGE 12) demonstrated no benefit of certain drugs conventionally used in therapy regimens of other BTC stages. In contrast, the positive outcome of the large UK BILCAP phase III trial initiated the use of capecitabine as an adjuvant therapy for resectable BTC patients, which is now standard of care and the control arm for newer studies.^59^^,^^61^^,^^62^ BILCAP compared adjuvant capecitabine with observation following surgery for BTC and found that capecitabine can improve OS according to the prespecified sensitivity and per-protocol analyses, although the primary endpoint of improving OS in the intention-to-treat population was not reached. The mOS in the prespecified per-protocol analysis was 53 months (95% CI 40 months-not reached) versus 36 months (95% CI 30-44 months) in the observation group (adjusted HR 0.75, 95% CI 0.58-0.97, *P* = 0.028).^61^

The Japanese JCOG1202:ASCOT phase III trial was published in January 2022 and reported a significantly longer survival of patients on S-1 therapy (an orally acting fluoropyrimidine derivative), when compared with surgery alone [HR 0.694 (95% CI 0.514-0.935, *P* = 0.008)].^6^^,^^63^ This has not, however, been reflected in guidelines yet.

Several trials assessing adjuvant therapy are still ongoing, e.g. the German phase II study ADJUBIL which investigates durvalumab and single dose tremelimumab (STRIDE regimen) in combination with capecitabine or without capecitabine and has recently finished recruiting patients in Germany.^64^

There are also multiple trials for neoadjuvant therapy in progress, for instance the Korean DEBATE trial examining durvalumab plus GemCis versus GemCis and the German GAIN phase III trial (estimated completion date Q4 2024) testing GemCis versus upfront surgery.^62^ Furthermore, the neoadjuvant German NeoTreme study examining the combination of durvalumab, GemCis, and single dose tremelimumab is currently recruiting.^65^

Although data from trials are lacking overall and there are some conflicting data, recently conducted trials point to adjuvant therapy being a valuable option for patients who underwent radical surgery for BTC and data from several comprehensive trials are expected to be published in the near future.^61^^,^^66^

### Advanced stage

Some 70%-80% of CCA patients are diagnosed at an advanced stage. They are not amenable to surgical resection and can therefore often only be treated palliatively.^67^ Data concerning mOS, median PFS (mPFS), and objective response rate (ORR) for the following paragraphs can be found in Tables 2 and 3.

**Table 2:** Studies concerning the advanced stage of biliary tract cancer.

	Study treatment	mOS (months)	PFS (months)	ORR (%)
ABC-02 phase III^68^	GemCis versus Gem	11.7 versus 8.1; HR 0.64 (95% CI 0.52-0.80; *P* < 0.001)	Median: 8.0 versus 5.0 (*P* < 0.001)	No data available
SWOG 1815 phase III^69^	GemCis versus GemCis plus nab-paclitaxel	12.7 versus 14; HR 0.93 (95% CI 0.74-1.19; *P* = 0.58)	Median: 6.4 versus 8.2, respectively; HR 0.92 (95% CI 0.72-1.16; *P* = 0.47)	34 versus 25 (*P* = 0.11)
PRODIGE 38 AMEBICA phase II^70^	GemCis versus mFOLFIRINOX	13.8 (95% CI 10.9-16.1) versus 11.7 (95% CI 9.5-14.2)	6-month PFS rate: 47.3% (90% CI 38.4-56.3) versus 44.6% (90% CI 35.7-53.7)	No data available
FUGA-BT phase III^71^	GemCis versus Gem plus S-1	13.4 versus 15.1; HR 0.945 (90% CI 0.78-1.15; *P* = 0.046 for non-inferiority)	Median: 5.8 versus 6.8	No data available
KHBO1401-MITSUBA phase III^72^	GemCis versus GemCis plus S-1	12.6 versus 13.5; HR 0.79 (90% CI 0.628-0.996; *P* = 0.046 stratified log-rank test)	Median: 5.5 versus 7.5; HR 0.75 (95% CI 0.577-0.970; *P* = 0.015)	15.0 versus 41.5 (*P* < 0.001)
TOPAZ-1 phase III^a^^73^^,^^74^	GemCis versus GemCis plus durvalumab	11.3 versus 12.9; HR 0.76 (95% CI 0.64-0.91)^74^	Median: 5.7 versus 7.2; HR 0.75 (95% CI 0.63-0.89; *P* = 0.001)^73^	18.7 versus 26.7^73^
KEYNOTE-966 phase III^5^	GemCis versus GemCis plus pembrolizumab	10.9 versus 12.7; HR 0.83 (95% CI 0.72-0.95)	Median: 5.6 versus 6.5; HR 0.87 (95% CI 0.76-0.99)	28.7 versus 28.7^75^

CI, confidence interval; Gem, gemcitabine; GemCis, gemcitabine plus cisplatin; HR, hazard ratio; mFOLFIRINOX, oxaliplatin, irinotecan, and infusional fluorouracil; mOS, median overall survival; ORR, objective response rate; PFS, progression-free survival.

a These data represent the updated mOS.^74^ Original mOS was 11.5 months versus 12.8 months; HR 0.80 (95% CI 0.66-0.97; *P* = 0.021).^73^

**Table 3:** Studies concerning second- and further line therapies of biliary tract cancer.

	Study treatment	mOS (months)	mPFS (months)	ORR (%)
ABC-06 phase III^76^	mFOLFOX versus ASC	6.2 (95% CI 5.4-7.6) versus 5.4 (95% CI 4.1-5.8); [adjusted HR 0.69 (95% CI 0.50-0.97; *P* = 0.031)]	No data available	No data available
NIFTY phase IIb	Irinotecan, fluorouracil, folinic acid versus fluorouracil, folinic acid	No data available	4.2 versus 1.7; HR 0.61 (0.44-0.86; *P* = 0.004)	No data available
ClarIDHy phase III^77^	Ivosidenib versus placebo	10.3 (95% CI 7.8-12.4) versus placebo 5.1 (95% CI, 3.8-7.6); HR 0.49 (95% CI 0.34-0.70; *P* < 0.001)	2.7 (95% CI 1.6-4.2) versus 1.4 (95% CI 1.4-1.6); HR 0.37 (95% CI 0.25-0.54; *P* < 0.0001)	No data available
FIGHT-202 phase II^78^	Pemigatinib	21.1 (95% CI 14.8-not reached)	6.9 (95% CI 6.2-9.6)	35.5 (95% CI 26.5-45.4)
PROOF phase II^79^	Infigratinib	No data available	5.8 (95% CI 4.3-7.6)	18.8
FOENIX-CCA2 phase II	Futibatinib	20.0	8.9	41.7
ReFocus phase I/II^80^	RLY-4008	No data available	No data available	88.2 (95% CI 63.6-98.5)
ROAR phase II^34^	Dabrafenib plus trametinib	14.0	9.0	51 (95% CI 36-67)
MyPathway phase IIa^17^	Pertuzumab plus trastuzumab	10.9	4.0	23 (95% CI 11-39)
KEYNOTE-158 phase II^81^	Pembrolizumab	23.5 (95% CI 13.5-not reached)	4.1 (95% CI 2.4-4.9)	34.3 (95% CI 28.3-40.8)
HERIZON-BTC-01 phase II^b^^16^	Zanidatamab	Still pending	Still pending	41 (IHC 2+/3+) versus 0 (IHC 0/1+)
SGNTUC-019 phase II^82^	Tucatinib plus trastuzumab	12: 53.6% (90% CI 36.8-67.8)	5.5 (90% CI 3.9-8.1)	46.7 (90% CI 30.8-63.0)
KCSG-HB19–14 phase II^83^	Trastuzumab plus FOLFOX	10.7 (95% CI 7.9-not reached)	5.1 (95% CI 3.6-6.7)	29.4 (95% CI 16.7-46.3)
HERB phase II^84^	Trastuzumab deruxtecan	*HER2*+: 7.1 (95% CI 4.7-14.6)	*HER2*+: 4.4 (95% CI 2.8-8.3)	*HER2*+: 36.4 (90% CI 19.6-56.1)
Lapatinib in advanced BTC (and HCC) phase II^85^	Lapatinib	5.2 (95% CI 3.3-∞)	1.8 (95% CI 1.7-5.2)	No data available
TAB phase II^86^	GemCis plus trastuzumab	9.95 (95% CI 9.25-10.66)	7.95 (95 % CI 6.84-9.06)	No data available

ASC, active symptom control; BTC, biliary tract cancer; CI, confidence interval; HCC, hepatocellular carcinoma; HR, hazard ratio; mFOLFOX, folinic acid, fluorouracil, oxaliplatin; mOS, median overall survival; mPFS, median progression-free survival ORR, objective response rate.

#### Systemic therapies—chemotherapy—immune checkpoint inhibitors and targeted therapies

**Figure 1:**
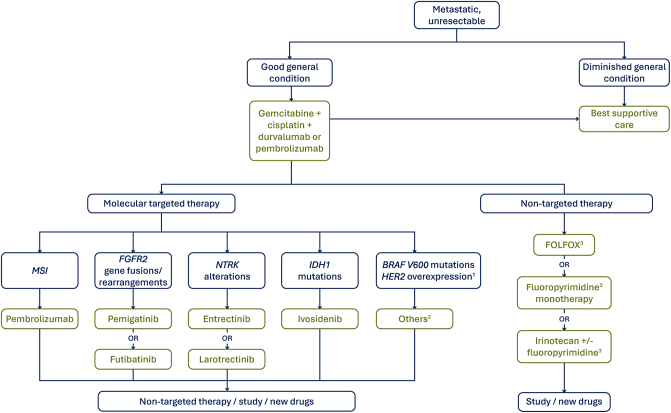
Treatment algorithm for biliary tract cancer in the advanced setting (modified after Sinn et al. 2024).^38^ BRAF V600, v-Raf murine sarcoma viral oncogene homolog B; FGFR2, fibroblast growth factor receptor 2; HER2, human epidermal growth factor receptor 2; IDH1, isocitrate dehydrogenase 1 mutation; MSI, microsatellite instability; NTRK, neurotrophic receptor tyrosine kinase. ^a^Targeted therapies for HER2 are under investigation, but not yet approved. ^b^Options for the off-label use: BRAF V600 – dabrafenib plus trametinib; HER2 – trastuzumab plus pertuzumab, neratinib, trastuzumab-deruxtecan, tucatinib plus trastuzumab, zanidatamab. ^c^Bevor starting a chemotherapy containing fluoropyrimidine, a dihydropyrimidine dehydrogenase (DPD) deficiency must be ruled out.

In unresectable or distant metastatic stage, systemic therapy with palliative intent is recommended.^35^ The individual treatment regimen is determined on the basis of molecular testing among other relevant factors such as the patient’s general condition, comorbidities, patient preferences, and toxicity of the planned regimen. The Onkopedia guideline recommends the testing of FGFR2, IDH1, NTRK, and MSI-H/dMMR in the palliative setting. As part of an off-label approach, the testing of BRAF V600 and Her2/neu can be considered.^38^ Detailed information on treatment pathways can be found in Figure 1.

#### Chemotherapy

Until recently, the standard regimen for advanced stage BTC consisted of GemCis only, which has proven effective in the phase III ABC-02 trial.^1^^,^^87^ ABC-02 showed a significant mOS advantage of GemCis versus Gem alone.^68^ Additionally, mPFS and tumour control (81.4% versus 71.8%, *P* = 0.049) were significantly increased in the GemCis group versus Gem-only group.^68^ Recent studies supporting GemCis as standard of care for advanced BTC are the phase III study SWOG 1815 and the phase II study PRODIGE 38 AMEBICA.^69^^,^^70^ Further information on these studies can be found in Table 2.

More than 10 years after publication of the UK ABC-02 and Japanese BT22 studies in 2010—which demonstrated superiority of GemCis over gemcitabine monotherapy for the treatment of BTC—the first-line chemotherapy regimen for BTC has remained unchanged.^68^^,^^88^^,^^89^ Although the administration of GemCis for palliative, first-line systemic therapy of unresectable or metastatic BTC is guideline-compliant, it still represents an off-label treatment in Germany.^35^ Consequently, there is a great medical need for effective, safe, and approved therapeutic options for the first-line systemic treatment of BTC.^90^

#### Immune checkpoint inhibitors

The ICI durvalumab in combination with GemCis followed by durvalumab maintenance therapy is the first immuno-oncology approach for the treatment of BTC. The TOPAZ-1 study demonstrated that durvalumab plus GemCis improved mOS, with an estimated 24-month OS rate of 24.9% for durvalumab (95% CI 17.9% to 32.5%) and 10.4% for placebo (95% CI 4.7% to 18.8%), prolonged PFS, and increased ORR compared with GemCis plus placebo.^73^ There were no new safety signals in addition to the established safety profile of durvalumab as monotherapy and consistent with the known safety profile of GemCis.^4^ Furthermore, time to deterioration in quality-of-life scores was unchanged when durvalumab was added to GemCis.^73^ An update to TOPAZ-1—5 months after the first results were published—showed that durvalumab plus GemCis led to longer mOS and improved OS rates (12 months: 54.3% versus 47.1%; 18 months: 34.8% versus 24.1%; 24 months: 23.6% versus 11.5%, respectively).^74^ This is further supported by data published in 2024 which demonstrated that the 36-months OS for durvalumab plus GemCis was 14.6% versus 6.9% for placebo plus GemCis.^91^ In all prespecified subgroups [e.g. sex, age, programmed death-ligand 1 (PD-L1) expression, disease status, primary tumour location, race, region, ECOG performance status], OS HRs favoured durvalumab combination therapy.^74^ These results further support the administration of durvalumab plus GemCis as new standard of care in patients with advanced BTC. Consequently, durvalumab plus GemCis was approved by the Food and Drug Administration (FDA) in September 2022 and by the European Medicines Agency (EMA) in December 2022. Additionally, recent results from the KEYNOTE-966 trial comparing pembrolizumab in combination with GemCis versus GemCis alone further validate the benefits of combining an ICI with chemotherapy in advanced BTC.^5^ KEYNOTE-966 reported an improvement in OS versus GemCis alone and prolonged median duration of response (DOR), but did not significantly improve mPFS or ORR versus GemCis alone.^5^ The clinically meaningful improvement in OS with pembrolizumab plus GemCis compared with GemCis alone was maintained after 36 months (OS rate of 13% versus 11%, respectively).^75^ Consequently, international guidelines recommend durvalumab or pembrolizumab plus GemCis as the preferred first-line treatment regimen for patients with unresectable, metastatic, or recurrent iCCA, eCCA, and GBC.^6^^,^^7^^,^^35^^,^^38^

Triplet regimens like mFOLFIRINOX (PRODIGE 38 AMEBICA), GemCis plus nab-paclitaxel (SWOG 1815), and GemCis plus S-1 (KHBO1401-MITSUBA) were taken into consideration as alternative chemotherapy regimens, but ultimately did not prevail in Western countries, partly due to either inferiority or non-superiority.^59^ GemCis plus S-1 showed survival benefits and a higher response rate (RR) over GemCis, however, and is a treatment option in Asian countries.^72^

#### Second- and further line therapies

Between 15% and 25% of first-line patients go on to receive second-line treatment after tumour progression.^1^ For those patients, no standard of care has been established as second-line therapy.^1^ The German S3 guideline recommends to offer a second-line therapy such as the chemotherapy regimen mFOLFOX (folinic acid, fluorouracil, oxaliplatin) after failure or intolerance to first-line therapy.^35^

The phase III UK ABC-06 trial showed that the addition of mFOLFOX improved mOS, when compared with active symptom control (ASC) alone.^76^ mOS was slightly longer for ASC plus mFOLFOX versus ASC alone. Additionally, 12-month OS rate [25.9% (95% CI 17.0% to 35.8%) versus 11.4% (95% CI 5.6% to 19.5%)] and 6-month OS rate [50.6% (95% CI 39.3% to 60.9%) versus 35.5% (95% CI 25.2% to 46.0%)] were longer in the mFOLFOX plus ASC arm compared with ASC alone.^76^ Researchers suggested mFOLFOX to be the new treatment standard for patients in second-line treatment of advanced BTC, which National Comprehensive Cancer Network (NCCN) guidelines reflect by elevating mFOLFOX to the preferred second-line treatment option.^92^ ESMO guidelines and German DGHO Onkopedia guidelines also recommend mFOLFOX as standard of care for second-line treatment after first-line GemCis.^6^^,^^38^ If intolerance occurs, fluorouracil plus irinotecan should be administered.

An alternative to the mFOLFOX treatment regimen was tested in the phase IIb Korean NIFTY study. PFS in the irinotecan plus fluorouracil and folinic acid group was significantly longer compared with fluorouracil and folinic acid alone in a second-line setting.^93^ ESMO guidelines describe the impact on OS as modest and on par with ABC-06.^6^ NIFTY represents an important study though, because it had a strong comparator arm with fluorouracil plus folinic acid. The phase II AIO NALIRICC trial showed that the addition of nanoliposomal irinotecan to fluorouracil plus leucovorin did not improve PFS or OS and was associated with higher toxicity compared with fluorouracil plus leucovorin.^94^ Furthermore, there is an ongoing discussion on whether it is reasonable to use mFOLFOX—which includes oxaliplatin—after cisplatin therapy. Especially in cases where GemCis was administered more often than the proposed original eight cycles, the use of oxaliplatin after cisplatin seems debatable.

For second- and further-line therapies, ESMO and German DGHO Onkopedia guidelines recommend specific approaches for genetic alterations after molecular profiling. The most frequent targetable alterations are *IDH1/IDH2* mutations, *FGFR2* fusions, *HER2/neu* overexpression, and *BRAF*^*V600E*^ mutations.^6^^,^^12^^,^^19^^,^^38^

During the phase III ClarIDHy trial, either ivosidenib (a first in class oral inhibitor of mutated *IDH1*) or placebo were administered to 230 chemotherapy-refractory, *IDH1*-mutant-positive patients. The mPFS (primary endpoint) was significantly improved in the ivosidenib arm compared with the placebo arm.^77^ After adjustment for a cohort of patients that crossed over from placebo to ivosidenib, there was a statistically significant improvement in mOS (secondary endpoint) as well.^95^ Accordingly, ivosidenib is now recommended by guidelines for CCA patients with *IDH1* mutations.^6^^,^^35^^,^^38^

The second most common genetic alterations to target—present in 13%-17% of iCCA patients—are *FGFR2* fusions and rearrangements. One of the first studies to assess a targeted drug for *FGFR2* fusions was the phase II, single-arm, open-label FIGHT-202 trial. Pemigatinib (an oral, selective inhibitor of *FGFR1*, *2*, and *3*) was administered to all patients with previously treated CCA in a locally advanced or metastatic setting.^23^^,^^78^ Patients were assigned to cohorts according to the status of their genomic alterations: *FGFR2* fusions/rearrangements, other *FGF/FGFR* gene alterations, or no *FGF/FGFR* gene alterations. ORR for the *FGFR2* fusions/rearrangements arm was 35.5% (95% CI 26.5% to 45.4%), disease control rate (DCR) was 82% (95% CI 74% to 89%), mPFS was 6.9 months (95% CI 6.2-9.6 months), and mOS was 21.1 months (95% CI 14.8 months-not reached).^23^^,^^78^ These results supported pemigatinib as a standard treatment of *FGFR2* fusion- or rearrangement-positive patients and led to its approval by the FDA and EMA. Genomic data acquired during the FIGHT-202 trial were also used to assemble a database of mutational profiles.^9^ Here, it was found that ∼45% of patients with CCA harbour targetable genomic alterations and that there is a diverse range of co-mutations. Through analysis of patients treated with pemigatinib, researchers found other primary and acquired mechanisms of resistance as well as targetable *FGF/FGFR* alterations.^9^ The FIGHT-302 study is recruiting now and compares first-line pemigatinib against GemCis for patients harbouring *FGFR2* rearrangements in the advanced CCA setting.^96^

Infigratinib is a pan-*FGFR2* inhibitor that was assessed in the phase II, single-arm, open-label PROOF study with *FGFR2* fusion-, mutation-, or amplification-positive patients.^97^ Only patients with *FGFR2* fusion achieved a response [ORR 18.8%; DCR 83.3%; mPFS 5.8 months (95% CI 4.3-7.6 months)]. In this highly specific group of previously treated CCA patients with *FGFR2* fusions, infigratinib showed a meaningful clinical activity.^97^ A subsequent study of infigratinib with a similar design reported an ORR of 23.1% (95% CI 15.6% to 32.2%), median DOR was 5.0 months (range 0.9-19.1 months), and mPFS was 7.3 months (95% CI 5.6-7.6 months).^79^ As a result of these findings, infigratinib was recently granted approval by the FDA for patients with previously treated locally advanced or metastatic CCA harbouring an *FGFR2* fusion or rearrangement.

Futibatinib is an irreversible *FGFR1-4* inhibitor, which was evaluated in a phase I dose-expansion study including patients with a broad spectrum of tumours harbouring *FGFR1-3* alterations.^98^ Futibatinib achieved an ORR of 13.7% in all tumours. In a subgroup analysis of *FGFR2* fusions/rearrangements-positive iCCA, ORR was 25.4%.^98^ Additionally, the phase II, open-label, single-arm FOENIX-CCA2 trial assessed futibatinib in *FGFR2* fusion/rearrangement-positive iCCA only.^99^ Here, ORR was 41.7%, DOR was 9.7 months, DCR was 82.5%, mPFS was 9.0 months, mOS was 21.7 months, and 12-month OS-rate was 72%.^99^ Researchers assumed the differences in ORR between the two studies can be traced back to low sample size and the ratio of patients with prior *FGFR*-targeted treatment.^98^ Further trials, e.g. the phase III FOENIX-CCA3 trial comparing GemCis to futibatinib in the first-line treatment of advanced CCA, are still ongoing but futibatinib was approved by the FDA in September 2022 and by the EMA in July 2023.^99^ Interestingly, futibatinib’s inhibitory activity even triggered a response in patients with an acquired resistance to prior *FGFR* inhibitors.^100^

Another point of action to enhance *FGFR* inhibitors and overcome resistance is via the inhibition of epidermal growth factor receptor (*EGFR*).^101^ Although small-molecule *FGFR2* inhibitors have proven efficacious in patients with CCA displaying *FGFR2* fusions and rearrangements, response rates are modest and resistance can develop rapidly, especially in patients with secondary *FGFR2* mutations.^102^^,^^103^ Polyclonal, secondary *FGFR2* mutations constitute a crucial resistance mechanism and should be analysed through molecular sequencing of ctDNA to promote the benefits of *FGFR* inhibitors.^104^ By inhibiting *EGFR in vivo*, response to *FGFR* inhibitors increased, while apoptosis and tumour regression were induced.^101^ Thus, there is potential for improving efficacy of and resistance to *FGFR* inhibitors through administration of *EGFR* inhibitors.^101^ No *EGFR* inhibitors, however, are currently approved for clinical use.

The recently conducted phase I/II ReFocus trial demonstrated the ability of the first highly selective *FGFR2* inhibitor RLY-4008 to overcome *FGFR* resistance mutations. Previous non-selective *FGFR* inhibitors, i.e. pemigatinib, infigratinib, futibatinib, were limited by polyclonal *FGFR2* resistance, so they achieved an ORR of around 20%-40% and DOR of around 5-9 months.^80^ The first results of the ReFocus study seem promising with an ORR of 88.2% (95% CI 63.6% to 98.5%) for patients who received the recommended phase II dose of RLY-4008.^80^ Another ongoing phase II trial is investigating the spectrum-selective multi-kinase inhibitor tinengotinib. Preliminary results show that overall DCR (complete response or partial response plus stable disease) was 90% (9/10) in *FGFR2* fusion/rearrangement patients, 100% in a *FGFR* primary mutation patient (1/1), and 71% (5/7) in *FGFR* wild-type patients.^105^ In conclusion, the approval of pemigatinib and futibatinib represents a breakthrough for *FGFR2*-positive CCA patients, which is reflected in a recommendation in ESMO and DGHO Onkopedia guidelines for patients who experience disease progression after more than one prior systemic therapy.^6^^,^^38^ Other guidelines for which pemigatinib was the only approved agent at the time of the guideline’s conceptualization, e.g. the German S3 guideline, may not yet mention futibatinib, but this is expected to change with future updates.^35^ New treatment options, however, are still needed for patients who harbour other genetic alterations, encounter reduced efficacy, and display resistance, or experience toxicity to the aforementioned drugs.^101^

*BRAF*^*V600E*^ mutations were addressed by the treatment regimen dabrafenib plus trametinib during the phase II, open-label, single-arm ROAR basket trial.^34^ Combination therapy showed promising results for *BRAF*^*V600E*^-positive patients as 51% (95% CI 36% to 67%) achieved an overall response, mPFS was 9.0 months and mOS was 14.0 months.^34^ Routine testing and—if positive—subsequent therapy with dabrafenib plus trametinib can be considered in therapy-refractory patients.

In patients with *HER2/neu* amplification, overexpression, or both a combination of pertuzumab and trastuzumab achieved an encouraging response rate over the course of the phase IIa, open-label MyPathway basket study.^17^ ORR, as the primary endpoint, was 23% (95% CI 11% to 39%), mPFS was 4.0 months, and mOS was 10.9 months.^17^ The multicentre, open-label phase II study DESTINY-PanTumor02 investigated trastuzumab deruxtecan (T-DXd) in seven tumour cohorts expressing *HER2/neu*, one of them being BTC. The ORR, as the primary endpoint, was 37.1% (95% CI 31.3% to 43.2%) in all patients, with responses observed in all cohorts. The mPFS was 4.6 months (95% CI 3.1-6.0 months) for the subgroup of BTC. Within this group, mPFS was 4.2 months (95% CI 2.8-6.0 months) for the immunohistochemical *HER2/neu* status 2+ (IHC2+) and 7.4 months (95% CI 2.8-12.5 months) for IHC3+.^106^ In the previously conducted phase II trial DESTINY-PanTumor01 the expression of *HER2/neu* was no predictor of response to T-DXd. The confirmed ORR (cORR) for all groups independent of the *HER2/neu* expression status was 29.4% (95% CI 20.8% to 39.3%), indicating efficacy also in *HER2/neu*-negative tumours with *HER2/neu* mutations.^107^ Other studies (HERB, SUMMIT) demonstrated a modest improvement in response rate for other agents targeting *HER2/neu* amplifications, overexpression, or both, but there is no FDA or EMA approved drug yet.^31^^,^^84^ The open-label phase II basket study SGNTUC-019 investigated the combination of tucatinib and trastuzumab in locally determined *HER2/neu* overexpressing or amplified and metastatic BTC after failed systemic therapy. The cORR was 46.7% (90% CI 30.8% to 63.0%), including 1 complete and 13 partial responses (*n* = 30). Median DOR was 6.0 months (90% CI 5.5 months-not estimable), DCR was 76.7% (*n* = 23; 90% CI 60.6% to 88.5%), and mPFS was 5.5 months (90% CI 3.9-8.1 months).^82^ The global open-label phase IIb study HERIZON-BTC-01 examined zanidatamab in patients with *HER2/neu*-amplified, locally advanced unresectable or metastatic BTC after gemcitabine-containing therapy. Patients were divided in two cohorts: cohort 1 for IHC 2+/3+ (*HER2/neu*-positive) and cohort 2 for IHC 0/1+. No responses were observed in cohort 2. In cohort 1, cORR was 41% with median DOR of 12.9 months (95% CI 5.95 months-not estimable). PFS and OS are still pending.^16^

In the phase II trial of the Korean Cancer Study Group, FOLFOX plus trastuzumab was evaluated as a second- or third-line treatment of HER2-positive BTC. mPFS was 5.1 months (95% CI 3.6-6.7 months) and mOS 10.7 months (95% CI 7.9 months-not reached).^83^ Trastuzumab and GemCis improved the 6-month PFS in HER2-positive, treatment-naive BTCs compared with historical data in the phase II study TAB.^86^

In *MSI-H/dMMR*-positive cancers, the prospective phase II KEYNOTE-158 trial demonstrated that the anti-PD-1 inhibitor pembrolizumab led to a clinical benefit.^81^ ORR was 34.3% (95% CI 28.3% 40.8%), mPFS was 4.1 months (95% CI 2.4-4.9 months), and mOS was 23.5 months (95% CI 13.5 months-not reached).^81^ Based on these data, pembrolizumab was recently approved for advanced, pretreated BTC with *MSI-H/dMMR*. At present, immunotherapy combined with chemotherapy according to TOPAZ-1 and Keynote-966 is already a new standard of care for the treatment of patients with advanced BTC in the first-line situation. Since immunotherapy is already available in the first line, the role of second-line pembrolizumab in patients with MSI-H/dMMR is less important.

In summary, there is an evolving number of targeted treatment options for second- and further-line treatment of patients with BTC and druggable alterations. Some are already approved by the FDA and/or EMA, while others are still in early studies or development.

## Future perspectives, upcoming biomarkers, early-stage therapies, and ongoing trials

After a decade with no major changes in first- and second-line treatment of BTC in the advanced setting, new treatment options are now emerging due to the rapidly evolving field of targeted therapies. Multiple new medications gained approval recently and with the addition of durvalumab to the GemCis regimen, a new valuable option for BTC patients is now available.

Furthermore, numerous studies on new drugs are still ongoing. Especially treatments targeting *FGFR2* and *IDH1/IDH2* have demonstrated effectiveness and are therefore at the centre of current research. Moreover, the targeting of *HER2/neu* (including amplification, overexpression, and activating mutation) is a topic of interest since *HER2/neu* can be found in all subtypes of BTC. In the adjuvant setting, there are many ongoing trials, for example, the ICI rilvegostomig combined with chemotherapy [capecitabine, S-1 (tegafur/gimeracil/oteracil) or GemCis] (ARTEMIDE-Bil01^108^), the two ICIs durvalumab and tremelimumab combined with or without capecitabine (ADJUBIL^64^) or a therapy targeting *FGFR2* fusion or rearrangement in combination with SBRT in the curative and adjuvant setting (PEARLDIFER^56^). Other trials investigate the combination of ICIs and standard of care GemCis chemotherapy in the neoadjuvant setting (e.g. NeoTreme, GAIN).^65^^,^^109^

For patients harbouring genetic alterations, the development of these new drugs represents a milestone, even though resistance to and toxicity of targeted therapies is an issue that needs to be addressed. Genetic alterations of patients should be identified during or before first-line therapy to plan further steps in treatment regimen. In the future, one of the most important tasks apart from developing new drugs will be identifying subgroups of patients with genetic alterations and matching them to the treatment option associated with the most favourable outcome. Consequently, biomarkers such as miRNAs and especially miR-221 harbour the potential to augment diagnosis, intervention, and prognosis of BTC. They show promising trial results and should be evaluated as upcoming biomarkers for BTC.^13^

In conclusion, a new wave of biomarker-driven targeted therapies and immunotherapies for BTC is on the horizon, which could bring major benefits to patients’ treatment outcomes and quality of life.
